# Platelets in Multiple Sclerosis: Early and Central Mediators of Inflammation and Neurodegeneration and Attractive Targets for Molecular Imaging and Site-Directed Therapy

**DOI:** 10.3389/fimmu.2021.620963

**Published:** 2021-02-19

**Authors:** Jacqueline M. Orian, Claretta S. D'Souza, Pece Kocovski, Guy Krippner, Matthew W. Hale, Xiaowei Wang, Karlheinz Peter

**Affiliations:** ^1^Department of Biochemistry and Genetics, La Trobe Institute for Molecular Science, La Trobe University, Melbourne, VIC, Australia; ^2^Department of Psychology and Counselling, School of Psychology and Public Health, College of Science, Health and Engineering, La Trobe University, Melbourne, VIC, Australia; ^3^Medicinal Chemistry, Baker Heart and Diabetes Institute, Melbourne, VIC, Australia; ^4^Atherothrombosis and Vascular Biology Laboratory, Baker Heart and Diabetes Institute, Melbourne, VIC, Australia; ^5^Department of Cardiometabolic Health, University of Melbourne, Melbourne, VIC, Australia; ^6^Molecular Imaging and Theranostics Laboratory, Baker Heart and Diabetes Institute, Melbourne, VIC, Australia; ^7^Department of Physiology, Anatomy and Microbiology, School of Life Science, La Trobe University, Melbourne, VIC, Australia

**Keywords:** platelets, multiple sclerosis, experimental autoimmune encephalomyelitis, neuroprotection, neuroinflammation, imaging, targeted therapy

## Abstract

Platelets are clearly central to thrombosis and hemostasis. In addition, more recently, evidence has emerged for non-hemostatic roles of platelets including inflammatory and immune reactions/responses. Platelets express immunologically relevant ligands and receptors, demonstrate adhesive interactions with endothelial cells, monocytes and neutrophils, and toll-like receptor (TLR) mediated responses. These properties make platelets central to innate and adaptive immunity and potential candidate key mediators of autoimmune disorders. Multiple sclerosis (MS) is the most common chronic autoimmune central nervous system (CNS) disease. An association between platelets and MS was first indicated by the increased adhesion of platelets to endothelial cells. This was followed by reports identifying structural and functional changes of platelets, their chronic activation in the peripheral blood of MS patients, platelet presence in MS lesions and the more recent revelation that these structural and functional abnormalities are associated with all MS forms and stages. Investigations based on the murine experimental autoimmune encephalomyelitis (EAE) MS model first revealed a contribution to EAE pathogenesis by exacerbation of CNS inflammation and an early role for platelets in EAE development via platelet-neuron and platelet-astrocyte associations, through sialated gangliosides in lipid rafts. Our own studies refined and extended these findings by identifying the critical timing of platelet accumulation in pre-clinical EAE and establishing an initiating and central rather than merely exacerbating role for platelets in disease development. Furthermore, we demonstrated platelet-neuron associations in EAE, coincident with behavioral changes, but preceding the earliest detectable autoreactive T cell accumulation. In combination, these findings establish a new paradigm by asserting that platelets play a neurodegenerative as well as a neuroinflammatory role in MS and therefore, that these two pathological processes are causally linked. This review will discuss the implications of these findings for our understanding of MS, for future applications for imaging toward early detection of MS, and for novel strategies for platelet-targeted treatment of MS.

## Introduction

Multiple sclerosis (MS) is a chronic autoimmune CNS disorder and one of the commonest causes of neurological disability in the young adult population worldwide (1). The disease is typified by the presence of lesions disseminated in time and space, characterized by inflammation, microglial reactivity, demyelination, axonal injury and neuronal loss (2). This widespread distribution of lesions accounts for the broad range of symptoms exhibited by affected individuals, including for example, sensory loss, fatigue, or difficulties with balance, mobility, bladder and bowel control, vision, speech and cognition. Women are more commonly affected than men and this difference is increasing in some areas of the world. North America and Europe have the highest prevalence and Asia and sub-Saharan Africa the lowest, but regardless of prevalence, the incidence of MS is rising worldwide (3, 4). MS is therefore a major health care burden for the individual affected, as well as for the respective health care system.

A number of treatment options are available for this condition, however, progress in this direction has been hindered by the limited understanding of the disease pathophysiology which is very complex. Historically, MS was viewed as a disease of white matter targeting principally myelin, due to the evidence of lesions and demyelination, considerably more prominent in white relative to gray matter (5). More recently, however, as a result of ground-breaking confocal microscopic investigations (6) and advanced magnetic resonance imaging (MRI) the neurodegenerative component of MS was brought to the forefront (7). This resulted in the revised view of MS as a global CNS disorder, with different, but partially overlapping pathophysiological mechanisms evolving over the disease trajectory. One type of mechanism is driven by classical inflammatory processes which are increasingly understood and addressed with current therapeutics (8). A second type begins in early disease development, is associated with diffuse neuro-axonal degeneration and becomes gradually more significant with age and disease duration. However, neurodegenerative mechanisms are poorly understood and remain untreatable so far (9–11). The literature suggests the existence of a soluble factor, produced by inflammatory cells, which induces neurodegeneration via stimulation of microglial reactivity, but the identity of this factor has remained elusive (12). On the other hand, the recent identification of platelets as new players in the development of EAE, a rodent neuroinflammatory disease which serves as an MS model (13–15), suggests that these anuclear components circulating in blood represent a credible candidate for this proposed pathogenic factor.

## The Changing View of MS

MS nearly always first manifests in young adults aged between 18 and 35 (3). In the majority of cases, the first indication of the disease consists of an acute episode of neurological dysfunction affecting one or several CNS regions, commonly the optic nerve, brainstem or spinal cord, associated with white matter lesion(s) identified by magnetic resonance imaging (MRI) (16). This presentation is defined as a clinically isolated syndrome (CIS). Confirmation of a clinical diagnosis of MS (clinically definite MS) is made with the use of gadolinium-based contrast agents (GBCA), which allows, in a single study, the identification of lesions disseminated in time (GBCA-enhancing lesions) and lesions disseminated in space, which constitutes the hallmarks of MS diagnosis (17).

The disease exists as a number of subtypes (18). The most common one is the relapsing-remitting form (RR-MS), involving over 80% of total MS cases. It is associated with earlier onset and a strong sex bias, affecting over three females to one male. RR-MS exhibits a disease course characterized by the occurrence of episodes of neurological dysfunction (or relapses) with or without recovery. This pattern is observed for 15–25 years, then followed in about 50% of RR-MS patients by transition to a progressive form characterized by worsening neurological decline without remissions. This is known as secondary progressive (SP)-MS. A less common form termed primary progressive (PP)-MS, is observed in about 10–15% of total MS cases where patients enter the neurodegenerative stage from the onset (19). This form more commonly first manifests in early middle age and affects females and males equally. Other rare forms exist, for example benign MS where patients are not symptom-free, but do not reach an irreversible stage, or MS with childhood onset beginning by 16 years of age (20). The relationship between these different MS forms is ambiguous, particularly in the absence of defined biomarkers for this disease (21, 22). It is unclear whether they represent distinct diseases, or clinical variants of a single disorder (23). Major questions that remain unexplained are the relationship between the two forms of progressive disease and mechanisms underlying the transition from RR to SP-MS.

The pathological hallmark unique to MS is the focal lesion resulting from primary demyelination and astrocytic scarring in a context of chronic inflammation (24). Focal demyelinated lesions are found in both white and gray matter and are associated with large-scale T and B lymphocytic infiltration resulting from major loss of blood brain barrier (BBB) function, together with oligodendrocyte death, axonal and neuronal injury and loss, leading to brain and spinal cord atrophy. Astrogliosis and microglial reactivity are also typical features of these lesions. These classic active lesions are particularly significant in white matter and in the early disease stage (in both acute and relapsing MS). Immunological and pathological studies, however, have revealed that inflammatory demyelination can result from a broad spectrum of mechanisms. Accordingly, profound heterogeneity has been demonstrated in lesions, whereby although all inflammatory infiltrates contain T cells and macrophages, the target can be either myelin or oligodendrocytes. Analysis of active lesions from biopsies (71 lesions from 51 cases) or autopsies (325 lesions from 32 cases) from early disease revealed multiple patterns (25), including Pattern I: lesions dominated by T cells and macrophages, Pattern II: lesions similar to Pattern I, but with accumulation of immunoglobulins and complement, suggestive of involvement of pathogenic antibodies, Pattern III: hallmarks of injury reminiscent of acute white matter stroke, characterized by reactive oxygen and nitric oxide radicals, or Pattern IV: severe oligodendrocyte degeneration in peri-plaque white matter, indicative of immune-mediated injury. In progressive stages, these active focal lesions become less prominent whilst increasing numbers of chronic active (slowly expanding plaques) and inactive lesions with reduced BBB damage are observed (2).

Gray matter lesions occur in multiple regions including deep gray matter nuclei, thalamus, hypothalamus, basal ganglia and spinal cord gray matter (26–30). They are already present from early disease stage, but with numbers and size increasing only moderately over disease progression. These lesions are also associated with perivascular inflammation, demyelination and microglial activation (2). However, the most significant aspect of gray matter pathology is in the form of cortical lesions, now identified as a major substrate of MS pathology (31, 32). This type of lesion occurs most commonly in the forebrain, cerebellum and hippocampus and is seen in biopsies or autopsies with disease duration of weeks to months, suggesting that they arise in early stages of disease development (33). These lesions result from a different type of inflammatory process, which is not coincident with large scale loss of BBB function, but with considerable meningeal accumulation of T and B cells. In their most severe form these accumulations can take the form of follicle-like structures containing distinct T and B cell sub-regions. The resulting meningeal infiltrates are associated with prominent microglial reactivity, the creation of a highly inflammatory parenchymal environment resulting in demyelination, together with major axonal and neuronal degeneration and extensive neuronal loss. However, the identity of the soluble inflammatory mediators which trigger microglial reactivity, remains unknown. These lesions are poorly detected by MRI and their extent and severity can only be fully gauged by post-mortem examination. They increase in number and size over time, thereby being more extensive in progressive disease, without apparent difference between PP and SP-MS.

In addition to the above, roles for CD8^+^ T-lymphocytes and B cells relevant to progressive MS have emerged, but remains ambiguous. CD8^+^ T-lymphocytes are the most abundant inflammatory immune cell sub-type in lesions, but are also associated with diffuse infiltration, active demyelination and slow accumulation of axonal damage in normal appearing gray and white matter, which also contribute to brain atrophy. B cells functions include antigen presentation, T cell activation and antibody-production (34–37). Furthermore as mentioned above, they become a core component of follicle-like structures, which eventually, may lead to compartmentalization of a B cell population independent of the peripheral B cell pool (38). It has been suggested that follicle-like structures develop during the RR phase as a result of recurring inflammatory activity and have been identified in SP-MS, in close association with gray matter lesions (39), thereby potentially acting as a source of antibodies and other pro-inflammatory components which can contribute to demyelination and neurodegeneration in the progressive stage.

The predominance of lesions, especially in white matter drove the early view of MS as a white matter disease. This plaque-centered approach to pathophysiological investigations, dramatically challenged by the identification of concurrent but apparent differential pathophysiological processes in different CNS regions and different disease stages, has been replaced by the view that there are two types of inflammation in MS, which are related but partly independent (2). The first type, associated with BBB breakdown and lymphocytic infiltration, is typical of the classic active demyelinated plaque. The second type is associated with meningeal accumulation of lymphocytes, absence of BBB breakdown, but severe demyelination and diffuse neuro-axonal degeneration. It is hypothesized that in the latter case, microglial responses to meningeal soluble factor(s) play a major role in neurodegeneration. The two types of inflammation occur in both RR and progressive disease forms. Future studies, therefore will need to focus on two key questions, namely: do the two types of inflammation reflect immune responses to different target antigens and what is the identity of the soluble factor which drives cortical demyelination and neurodegeneration?

## A Role for Platelets in MS and EAE

### The Non-hemostatic Functions of Platelets

Platelets are anuclear cells circulating in blood, historically associated with a role in hemostasis and more recently, with vascular inflammatory disorders and also cancer (40). They are derived by budding of megakaryocytes in the bone marrow and are unique in terms of their abundance (with a normal range of 150–400 × 10^9^/L in humans), small size (2–3 μm in diameter) and rapid turnover (with a lifespan of 8–9 days). Under laminar flow conditions, they travel along the endothelial cell layer lining the blood vessel wall, thereby enabling immediate recruitment and local activation at the site of injury, in response to physical damage and/or invading pathogens. They are also characterized by three types of secretory compartments, namely the α granules, dense granules and lysosomes, as well as a complex membranous system, known as the open canalicular system. Together, these structures allow the storage of polypeptides in precursor or processed form and rapid release upon platelet activation (41). Platelet products have autocrine or paracrine functions, essential to activate platelet aggregation and trigger coagulation cascades fundamental to platelet hemostatic functions in pathogen surveillance and wound healing (42, 43).

A growing literature has highlighted previously unsuspected, non-hemostatic roles for these cells in inflammation and immunity. It was commonly believed that because platelets are devoid of nuclei, they would not synthesize new proteins and that their protein content was endowed from megakaryocytes upon platelet budding. It has since been demonstrated that they possess abundant mRNA, as well as microRNA and non-coding RNA and all the elements of the transcriptional machinery required for protein synthesis (42). Studies integrating proteomic and genomic studies of platelets from healthy human donors revealed ~3,000 distinct mRNA species (44–47). This number illustrates the diverse repertoire of mediators, as well as mechanisms via which platelets modulate inflammatory functions. A number of the most abundant transcripts represent proteins already known to be produced in platelets, including adhesive proteins, coagulation factors, proteoglycans, immunoglobulins, proteases and protease inhibitors (48). However, such investigations also revealed expression of molecules with immunological functions. It is the discovery of these components that led to a paradigm change and the appreciation of the role of platelets in the continuum of immunity, namely in bridging innate to adaptive immunity. These components include for example, Toll-like receptors (TLR), a family of pattern recognition receptors, that identify and respond to conserved microbial pathogen-associated molecular patterns. To date TLR 1, 2, 3, 4, 6, 7, and 9 have been implicated in platelet responses (49). Platelets also express a wide range of cytokines, chemokines and their receptors. These molecules are central to inflammation by signaling leukocyte differentiation, migration and infiltration. Among the most potent molecules of that class are the cytokine IL-1ß, a key regulator of inflammatory responses, and others, originally known as platelet “growth factors,” such as transforming growth factor-ß (TGF-ß) and platelet-derived growth factor (PDGF) with mitogenic properties. Platelet chemokines include CCL5 (RANTES), CCL3 (macrophage inflammatory protein-1α), and CXCL4 (PF4) (50). There was agreement among profiling studies of a high level of concordance between detectable proteins and their mRNA transcripts, although differences were observed between levels of individual message and protein. Messages for about 30% of proteins could not be detected, which could be accounted for by differences in half-lives of mRNA and their corresponding products, paracrine delivery of mRNAs (as well as microRNA) to target cells, proteins carried over from megakaryocytes, or proteins scavenged from plasma, such as albumin and fibrinogen (42, 46).

In addition, platelet mediators include lipid components and indeed, platelets are primary lipid carriers in the circulation (51). Examples include the thromboxane A2 (TXA2; a metabolite of arachidonic acid) and platelet activation factor (PAF). TXA2, notable for its half-life of 30 s, is produced by activated platelets and acts in an autocrine and paracrine manner by stimulating activation of new platelets and promoting platelet aggregation, resulting in platelet shape change and degranulation. PAF is a small phospholipid signaling molecule (acetyl-glyceryl-ether-phosphorylcholine) on the platelet surface with pro-inflammatory and vasoactive properties, also produced by neutrophils, monocytes, endothelial cells, and neurons. PAF causes both platelet and neutrophil adhesion and activation and platelet synthesis of IL-1ß and elevated PAF levels have been identified in RR-MS (52, 53). Thus, platelets can both generate and respond to signals at early time points in the inflammatory process.

A further mechanism of platelet action is via the use of signaling molecules anchored on the plasma membrane and mediating cell-cell interactions. This provides the advantage of signaling localized to the site of injury, while co-incidentally maintaining precision of interaction between platelets and their cellular partners. Upon vascular injury, exposure of sub-endothelial matrix proteins is immediately followed by platelet tethering to the subendothelial extracellular matrix through multiple receptors, such as glycoprotein GPVI and the GP1b/IX/V complex, together with integrins αIIbß3 (GPIIb/IIIa), α2ß1, α5ß1, and α6ß1, thrombin receptors, P2Y12 receptors, and thromboxane receptors (50). In addition to integrins which promote platelet aggregate formation, platelets express molecules facilitating their interactions with endothelial cells and leukocytes including platelet endothelial cell adhesion molecule 1 (PECAM-1), intercellular cell adhesion molecule 2 (ICAM-2) and junctional adhesion molecules (JAM) which belong to the Ig superfamily. These receptors mediate morphological changes, activation, adhesion and aggregation. Platelet activation causes release of signaling molecules from granules causing recruitment and activation of additional platelets resulting in clot formation (54). Adherent platelets release large amounts of P-selectin from α granules to the surface of activated platelets, which recognizes P selectin glycoprotein 1 (PGSL1) on target cells, for example neutrophils. Platelets are also a major source of the bioactive modulator CD40 ligand (CD40L) a transmembrane protein belonging to the TNF-α family. CD40L binds its cognate receptor CD40, which is widely expressed, resulting in the activation of monocytes (dendritic cells and macrophages), B and T cells and endothelial cells (54).

### An Association Between Platelets and MS/EAE

#### Early Evidence of Platelet Involvement in MS

Historically, there has long been a postulate of a role for platelets in MS (55). Such evidence first emerged with reports by Putman (56, 57), suggesting a role for venule thrombosis in CNS demyelination. Subsequently, multiple studies demonstrated increased platelet adhesiveness in MS, relative to other neurological disorders, both in the percentage of patients exhibiting abnormalities in adhesiveness and in the extent of the differences. For example, a study of 60 MS patients and 12 healthy subjects was reported by Wright et al. (58), where MS patients were further classified as stationary, fluctuating or exhibiting acute exacerbation. Estimations of platelet adhesion indices revealed that levels of adhesiveness paralleled the clinical activity of the disease, whereby more than 80% of patients in the fluctuating group and over 90% of those in the group with acute exacerbation showed abnormal platelet adhesiveness, compared with 20% of patients identified as stable. Furthermore, the highest adhesive index was found in the group exhibiting exacerbation. These findings have since been repeatedly confirmed by other investigators (59), but additionally, have been shown to coincide with biochemical abnormalities in the form of alterations in lipid composition of platelet membranes and serum, notably reduced cholesteryl linoleate (60, 61), a quantitatively minor cholesterol ester in platelets (62). A significant relationship between reduced cholesteryl linoleate levels, platelet adhesiveness and level of disease activity was established. In this context, imbalances in lipid composition are now associated with the initiation and progression of CNS disorders, such as MS, Alzheimer's disease, and Parkinson's disease (63). Consequently, lipidomic profiling to identify changes in plasma levels or plasma/CSF ratios are increasingly being explored as an approach for biomarker discovery of pathological pathways in these conditions (64). Given the consistent observation of platelet adhesiveness in MS, it would be of interest to extend these lipidomic and biomarker studies to platelets.

#### Ultrastructural Modifications and Alterations in Membrane Constituents

The above biochemical abnormalities are consistent with changes of platelet ultrastructure in MS patients, documented by scanning electron microscopy, in the form of pseudopodium formation, aggregation and lysis, which are all markers of platelet activation. The generation of pseudopods is associated with cytoskeletal collapse, fusion of granule membranes with surface-connected membranes of the open canalicular system or with the plasma membrane and transfer of granule contents to the platelet surface. Activated platelets are characterized by surface expression of P-selectin, which is critical to platelet-leukocyte interactions. Thus, further evidence of platelet activation in MS was provided by the demonstration of significant elevation of P-selectin in a study which compared 33 treatment-naïve, clinically stable RR-MS patients, with 92 control subjects using flow cytometry (65). The same study also identified significantly elevated platelet-associated IgM but not IgG. This IgM may represent autoimmunity, because anti-phospholipid antibodies in MS are predominantly of the IgM sub-class (65). Non-organ-specific anti-phospholipid (aPL) antibodies are recognized markers of increased coagulation activity and have been investigated particularly in the context of intravascular thrombosis. In MS, higher aPL antibody levels relative to healthy controls have been reported in both RR- and SP-MS (66) and associated with more severe clinical disease and MRI-identified disease progression. APL antibodies are predominantly directed against phosphatidylethanolamine, cardiolipin and ß2-glycoprotein 1 (a multifunctional apolipoprotein which binds cardiolipin) (67). Alternatively (or additionally), this IgM may originate from immunoglobulins normally stored within platelets, since chronic platelet activation has been reported to cause externalization of immunoglobulins normally stored within platelets. Surface expression of IgM may be part of an opsonisation mechanism which sensitizes platelets for destruction by complement (48). The complement system is part of the innate immune system and consists of a complex array of circulating proteins, which identify and kill target cells via cascades of proteolytic conversions of zymogens to their active forms. Platelets express multiple receptors for complement and are very sensitive to complement-mediated attack, which has the effect of increased procoagulant activity (67).

Given the evidence of alterations of platelet membranes, attention also turned to membrane-bound components that regulate mechanisms modulating platelet aggregation. A study of 20 RR-MS patients and 20 healthy subjects (68) examined changes in the enzymes ectonucleoside triphosphate diphosphohydrolase (NTPDase [CD39]), ectonucleotide pyrophosphatase/phospho-diesterase (E-NPP), 5′-nucleotidase and adenosine deaminase (ADA), known to modulate platelet aggregation via regulation of extracellular adenine nucleotide levels. Extracellular adenine nucleotides ATP, ADP and AMP (in particular ADP) are potent promoters of platelet aggregation, while their nucleoside derivative adenosine is a potent inhibitor of this process. Therefore, the platelet aggregation status is regulated by the equilibrium between adenine nucleotides and adenosine by the combined activity of these four enzymes. This investigation identified significant reduction in hydrolysis of ATP, ADP and AMP associated with reduced activity of NTPDase, 5′-nucleotidase, E-NPP and ADA in MS patients relative to normal controls. These data suggest the presence of a pro-inflammatory/pro-thrombotic milieu in MS by the absence of nucleotide hydrolysis, ADP accumulation and decreased adenosine production.

#### Evidence of Platelet Products in Plasma of MS Patients

It is therefore not surprising that as a result of platelet activation, significant changes in levels of plasma platelet-specific components have been reported. These include for example the α granule components soluble P-selectin (69), as well as ß-thromboglobulin (ß-TG) and PF4 (70), with PF4 levels being shown to correlate with disease severity. In addition, platelet microparticles (MP) have been investigated in MS (64). MP are a heterogeneous population of small vesicles between 100 nm and 1 μm in diameter, released by cells under physiological and pathological conditions. They are generated by budding of the plasma membrane and the cargo they carry includes proteins, lipids, miRNA, non-coding RNA and cell surface receptors and antigens. Many of these components function as bioactive molecules, hence the identification of microparticles as vectors for intercellular communication, mediating target cell activation, phenotypic modification, and reprogramming. A multitude of pathologies including inflammation and autoimmune diseases have been associated with significant increase in MP release and since these particles express markers pertaining to their cells of origin, there is considerable interest in their potential as disease biomarkers. Platelet MP (PMP) are the most abundant ones in the circulation under homeostatic conditions. There have been multiple reports of a relationship between elevated PMP in the circulation and MS. In a flow cytometry study of 95 patients (including 12 CIS, 51 RR, 23 SP, 9 PP cases) and 49 healthy subjects Marcos-Ramiro et al. (71) demonstrated that significantly increased PMP levels relative to healthy controls were associated with all clinically definite MS forms. PMP levels in CIS patients were elevated but this increase was not significant. Therefore, this study concluded that platelet dysfunction manifests when patients are definitely progressing. Of interest was the additional finding that endothelial cell-derived MP were significantly increased in confirmed MS cases, as well as CIS cases, suggesting platelet activation to be secondary to endothelial cell damage. In a separate study, Sáenz-Cuesta et al. (72) identified elevated PMP in RR-MS, untreated and treated patients and furthermore demonstrated that numbers of circulating PMP, in particular, are indicative of treatment effect and clinical status in MS. Thus, the highest PMP levels were found during disease exacerbations while untreated MS patients also showed significantly higher PMP compared with controls. Treatments with IFN-ß, or the α-4-ß-1 integrin antibody blocker natalizumab was associated with higher MP levels, including PMP. On the other hand, this study showed that SP-MS patients exhibited no significant difference in PMP from healthy controls. These data were interpreted as being suggestive of a relationship between rising levels of platelet shedding and periods of active inflammation. The differences between the Marcos-Ramiro and Saenz-Cuenza may be related to methodological approaches, for example in the use of different markers for flow cytometric sorting of microparticle subsets. Additionally, they may be influenced by differential treatment effects between patient cohorts, since drugs, such as IFN-ß, or natalizumab are associated with increased PMP (73). The basis for the rise in PMP with drug treatment is still unclear, but may be a secondary effect of blockade of lymphocytic entry into the CNS and increasing blood lymphocytes and lymphocyte-derived MP. Nonetheless, despite the differences between studies in the PMP status in SP-MS, there is an overall consensus that platelets increase PMP generation during periods of exacerbations, suggesting a pathological function for this phenomenon (73).

In the study of Sheremata et al., it was found that P-selectin-positive PMP were capable of binding to PSGL-1 and PECAM-1 on lymphocytes by increasing levels of integrins, such as VLA-4, resulting in increased lymphocyte binding to the endothelium. Furthermore, PMP cargo was shown to contain PAF (48, 74). However, PAF is produced by a variety of cells involved in host defense and the coordinated secretion of PAF would enhance the opening of the BBB, since disruption of endothelial junctions is the most prominent effect of PAF. This evidence suggests that PMP serve as immunomodulatory agents engaged in the propagation of inflammation (74). To date however, there is still insufficient evidence to determine whether PMP are suitable as MS biomarkers.

#### Platelet-Specific Markers Found in MS Lesions and Normal Appearing White Matter (NAWM)

Platelets were shown to cross the BBB via the damaged vascular basal lamina, rather than the inter-endothelial cell junctions *in vivo* (75). Evidence of platelet-derived products has been demonstrated within MS lesions, or around lesions (that is in NAWM). In a study by Han et al. (76) proteomics analysis of laser-microdissected lesions including acute plaque, chronic active plaque and chronic plaque, proteins of the coagulation cascade, such as tissue factor and protein C were identified in chronic active plaques. The significance of this finding is that these platelet-related components are associated with an active stage of the disease. In a separate study the same laboratory identified the platelet-specific glycoprotein GPIIb (CD41) in chronic plaques (77).

Studies focused on the relationship between platelets and CNS demyelination demonstrated that fibrinogen is abundant in NAWM. Fibrinogen (plasma and platelet-derived) is emerging as a significant mediator of inflammation and potentially, a trigger of early lesion formation in MS (78). Fibrinogen is a 340-kDa multimeric glycoprotein that has critical functions in vascular hemostasis. Although fibrinogen normally circulates in plasma at concentrations approximating 3 mg/ml, its levels can exceed 7 mg/ml during inflammatory responses. At sites of inflammation, endothelial cell retraction permits extravasation of fibrinogen, leading to its extravascular deposition as mixed fibrin/fibrinogen polymers. Fibrinogen is known to promote innate immune activation, thereby driving local inflammation. Post-mortem studies have reported extensive fibrinogen deposits around blood vessels, not only in active and chronic MS lesions, but significantly in pre-active lesions, namely prior to inflammatory infiltration and demyelination.

#### Recent Novel Insights From EAE-Based Investigations

##### The EAE Model and Its Application to MS Research

EAE is a neuroinflammatory disease induced in susceptible species, which has been used as an MS model for several decades (13–15). It is generated by active immunization with CNS antigens, including spinal cord homogenate, purified myelin proteins, or their immunodominant epitopes, most commonly mice, rats, and non-human primates (13, 79). Depending on the mouse/rat strain and antigen combinations different clinical profiles can be generated (80, 81), but currently, the variant generated by the peptide containing amino acids 35–55 of the myelin oligodendrocyte glycoprotein (MOG) in the C57BL/6 mouse strain has taken the most prominent place in EAE-based investigations (82). Alternatively, the disease can be provoked by passive immunization with encephalitogenic CD4^+^ T cells isolated from draining lymph nodes of actively immunized donor mice into syngeneic animals.

Common symptoms include ambulatory difficulties, impaired balance, bladder and bowel dysfunction, as well as behavioral deficits (83, 84). The pathology of EAE consists of meningeal and perivenous inflammation, dominated by T cells and macrophages, associated with severe and widespread microglial and astrocytic reactivity. Axonal injury and neuronal loss are additional features of disease pathology, beginning early in disease development (85). Of interest is the observation that depending on the genetic background of the host and immunization regimen, EAE lesions are most reminiscent of Types I and II MS lesions (25). These clinical, histological and immunopathological hallmarks reminiscent of MS, together with the potential to access a wide range of genetically modified mouse lines, have made EAE an attractive experimental model to gain insights into MS immunopathological mechanisms and validate candidate MS therapeutics.

However, although MS and EAE share multiple common pathological mechanisms, they are distinct diseases (86–89). The model has been criticized for discrepancies with MS from the genetic perspective and because of its partial recapitulation of MS (13–15), but also significantly, for its lack of reliability in prediction of the efficacy of candidate MS therapeutics (90). On the other hand, there is no doubt that EAE has provided valuable proof of concept for mechanisms of immune-mediated injury (89, 91). A classic example is the differentiation between the roles of CD4^+^ and CD8^+^ T cells in CNS autoimmunity. Originally thought to be the major drivers of the inflammatory process, CD4^+^ T cells now appear to be involved in the initiation of the immune response rather than in the effector stage of brain inflammation. CD4^+^ T cells are outnumbered by CD8^+^ T cells in the ratio of 1 to 10 in MS lesions and data suggest that CD8^+^ T cells proliferate in response to myelin antigens, subsequently trafficking to sites of inflammation. Using MOG_35−55_-induced C57BL/6 mice, it was shown that MOG_37−46_ is a minimal peptide capable of inducing specific a CD8^+^ T cell response, thereby supporting the notion of a major role for CD8^+^ T cells in active tissue damage (92, 93). A second example is the identification of the Th17 subset, characterized by expression of the cytokines IL-17A, IL-17F, IL-21, and IL-22. Studies, mainly based on the use of the EAE model, have identified the multistep differentiation of Th17 cells including the pro-inflammatory cytokines driving this process, together with Th17-derived products which attract T cells and myeloid cell populations into lesions. Additionally, IL-17 acts directly on endothelial cells by induction of reactive oxygen species. Thus, whereas EAE was thought to be a prototypical Th1 driven autoimmune disease, it is now clear that Th17 cells play a more critical role in disease initiation than Th1 cells (93–95).

Therefore, whilst EAE recapitulates a limited number of facets of MS, no other currently available model demonstrates pathological features reminiscent of MS, or the capacity to address the autoimmune response. The consensus is that if used rationally, namely by recognizing the limitations of the model in the experimental design, EAE still represents a useful tool to elucidate specific mechanisms underlying MS pathophysiology and allow repair and neuroprotective strategies to be explored (96–98).

##### The Critical Role of Platelets in EAE Development

Overall, the sum of the evidence supports the concept that platelets are chronically active in MS and that their involvement begins early, since a number of these changes have even been identified at the CIS stage. Novel insight into the relationship between platelets and the development of neuroinflammation was provided by a series of EAE-based studies from several laboratories. Firstly, in a series of elegant experiments Langer et al. (99) demonstrated the presence of platelets in the mouse CNS via detection of the platelet-specific marker CD41. Subsequently, they demonstrated a beneficial effect of platelet depletion with anti-mouse platelet serum on EAE severity and progression, when platelets were depleted in the inflammatory phase of the disease. This was confirmed by evidence of reduced microgliosis, together with decreased spinal cord inflammatory components CCL2, CCL5, CXCR4, and IL-1ß and decreased axonal injury and demyelination. Furthermore, targeting of specific platelet components involved in platelet responses also ameliorated EAE. This was achieved by administration of blocking Fab to GP1bα, a component of the GP1b/IX/V complex, or Fab to the major platelet adhesion receptor GPIIb/IIIa. Importantly, this study also identified platelets in MS post-mortem tissue. These data, therefore, provided the first strong evidence that platelets contribute to the pathogenesis of EAE by promoting CNS inflammation.

In independent studies, Sotnikov et al. (100) identified CD41^+^ platelets/platelet aggregates directly associated with neuronal cell bodies and astrocytes from as early as day 6 post-EAE initiation, co-incident with a 25-fold increase in PF4 expression. They demonstrated that this occurs via sialated gangliosides in lipid rafts, specifically the gangliosides GT1b and GQ1b, confirmed by the use of mice genetically deficient in sialyltransferase ST3Gal-V (a synthase of GM3 ganglioside). This resulted in significant reduction in EAE symptoms, together with CD4^+^ T cells, lymphocytes and macrophages both in CNS tissues and in the circulation. The implications of these findings are that they demonstrated the presence of activated platelets in the CNS parenchyma and their direct association with CNS cells.

Our own studies began with mapping of platelet accumulation from day 0 of EAE to characterize the relationship between platelet changes and the development of inflammation. In D'Souza et al. (101) and Kocovski et al. (84) platelet accumulation in the circulation and its timing were identified from 3 days post-induction, peaking at about 7 days and remaining elevated over the rest of the disease course. This was distinctly earlier than the accumulation of autoreactive T cells in blood, lymphoid tissues and the CNS, which were only detectable beginning from 11 days after induction. Platelet entry into the CNS was also identified ahead of that of CD3 cells using a qPCR approach, but different outcomes were documented between white and gray matter. In white matter platelets were found to be disseminated throughout the tissue, while in gray matter they were closely associated with neurons. Both studies showed intimate platelet-neuronal associations in the spinal cord, retina (associated with retinal structural abnormalities) and hippocampus (a region associated with emotion, cognition and memory) immediately following platelet entry and at times preceding autoreactive T cell accumulation. Significantly, the efficacy of platelet depletion was directly related to the timing of platelet accumulation: thus, when depletion was performed only over the period preceding the peak of platelet accumulation, or initiated in the chronic stage of disease, the beneficial effect of treatment was reduced. On the other hand, when platelet depletion was initiated from the peak of accumulation, treatment eliminated parenchymal T cell accumulation, EAE development and the generation of a pro-inflammatory environment in the CNS. A functional relationship was confirmed when it was shown that platelet accumulation is associated with anxiety-like behavior and that this effect is reversed with platelet depletion.

An understanding of mechanisms underlying platelet effects on neuronal and glial cell functions is emerging, although some of these mechanisms have to be inferred from data derived from experimental models more relevant to other neurodegenerative conditions. Firstly, the study by Sotnikov et al. (100) establish a new role of platelets by demonstrating that these elements directly recognize sialated gangliosides in lipid rafts on the surface of neuronal processes and end feet of astrocytes and that these interactions are essential for EAE development. Gangliosides are commonly found in many tissues but are most abundant in the brain; however only gangliosides GT1b and GQ1b were recognized by platelets, highlighting the specificity of these interactions (100). The recognition of these sialated gangliosides by platelets involved principally P-selectin, consistent with the established pro-inflammatory role of this component. These data therefore, identify lipid rafts of astrocytes and neurons as new ligands within the CNS that are recognized by platelets, thereby suggesting a mechanism for platelet-CNS cell communication. In separate studies, using a model of traumatic brain injury (102), the same group showed that interaction of platelets with neuronal lipid rafts occurs within minutes of injury, leading to immediate release of platelet-derived factors, via platelet degranulation and PMP shedding. Degranulation was associated with the release of neurotransmitters, such as serotonin (5-HT), from dense granules. On the other hand PMP production results in surface localization of PAF (74). In this context, however, platelet-neuron association stimulated neuronal activity, increased neuronal survival near the site of injury and promoted the formation of new dendritic spines on these cells, demonstrating an alternative role for platelets in synaptic plasticity. It is of interest that similar glycolipid structures have also been documented on neural precursor cells (103, 104), implying that platelets may directly communicate with neural precursor cells via a receptor-mediated interaction.

A study aimed at identifying signals responsible for early response to CNS injury (105) demonstrated platelet effects on oligodendrocyte precursor cells (OPC). First, damage to the BBB was caused by microinjections of vascular endothelial growth factor (VEGF) or lipopolysaccharide (LPS) in mouse or rat basal ganglia, followed by delivery of blood and blood components. Responses of glial cells were quantified by sub-type specific activation markers after 24 h in the injected side relative to vehicle-only contralateral side. Delivery of whole blood, platelets, or macrophages, as well as injury-related cytokines associated with macrophages and platelets, such as TNFα, TGFß, IL-1ß, and IFNγ, resulted in OPC activation while OPC mitogenic factors, including platelet-derived growth factor (PDGF) and fibroblast growth factor 2 (FGF-2), failed to produce a similar effect. As expected, these observations were associated with prominent microglial reactivity. The role of OPC is normally associated with repopulation of depleted oligodendrocytes following demyelination. The rapid response shown here suggests a platelet-mediated OPC injury response related to wound-healing in the brain.

In addition to PMPs, platelets release exosomes, which are 40–100 nm diameter, CD63^+^ extracellular vesicles, originating from endosomal multivesicular bodies and α-granules. Platelet exosomes carry principally (but not exclusively) α-granule components (106), including several neurogenesis-promoting molecules. Therefore, exosome release represents a potential additional route for platelet targeting of CNS cells in response to BBB disturbances.

**Figure 1:**
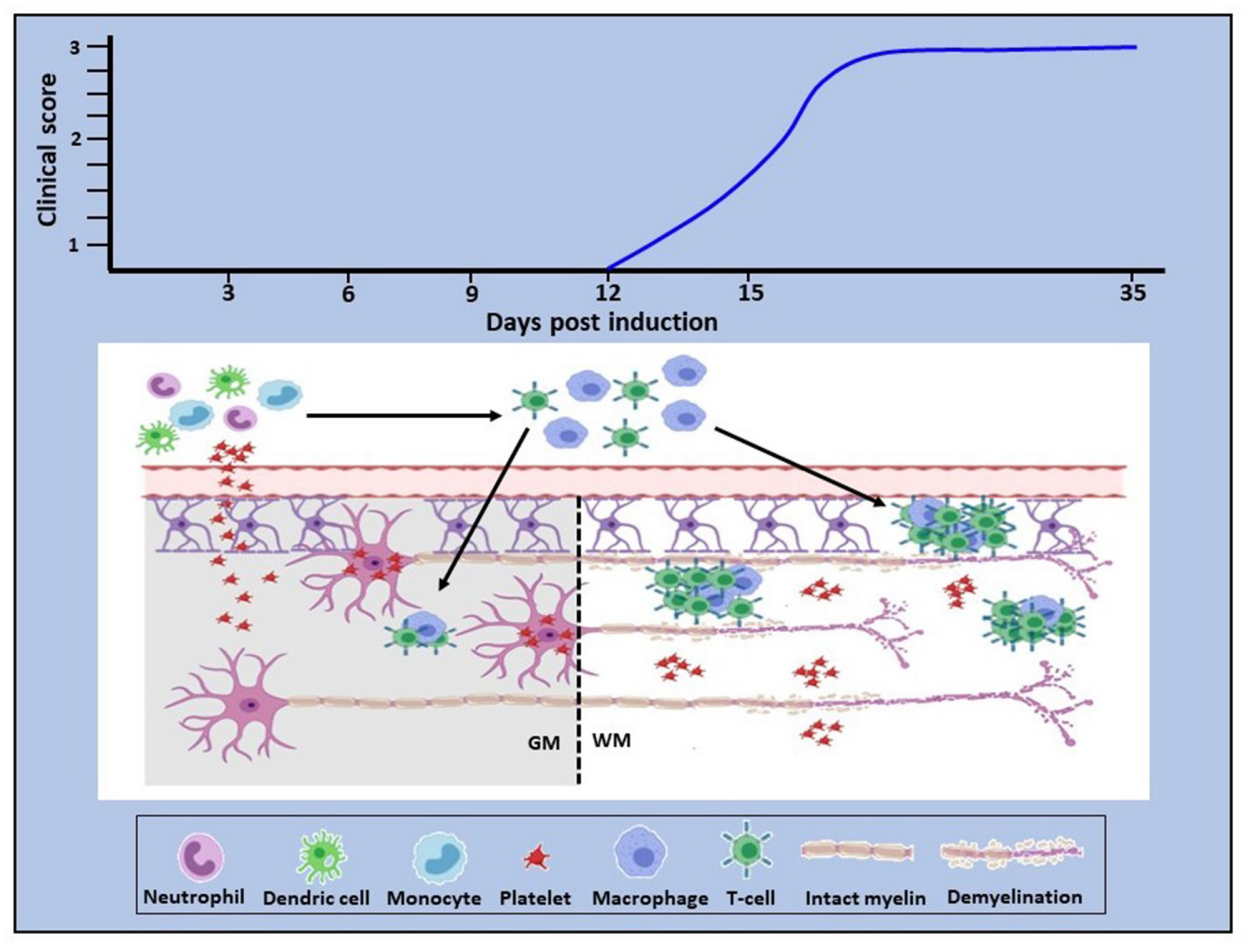
Proposed role of platelets in neuroinflammation. In EAE, platelet activation is an early event which is quickly followed by platelet infiltration into the CNS white and gray matter. In white matter they disseminate throughout the parenchyma, whereas in gray matter they target neurons (bottom panel). Concurrently, platelets drive the generation of autoreactive immune cells which become detectable by day 11–12 post-disease induction, corresponding to clinical onset (top panel). This evidence provides proof of concept that platelets are neurodegenerative as well as pro-inflammatory and that these two pathological processes are causally linked. Additionally, these data suggest that platelet targeting would represent an effective anti-inflammatory and neuroprotective therapeutic approach for MS. GM, gray matter; WM, white matter.

These recent findings, therefore, bring new insight into mechanisms underlying neuroinflammation. They demonstrate early platelet involvement in disease development which drives two distinct but related pathophysiological processes (Figure 1). First, platelets cross the BBB (and potentially other barriers, such as the blood-retinal barrier), become closely associated with and deliver pro-inflammatory products to neural (and potentially glial) cells via lipid rafts, PMP and exosomes, thereby using the full armamentarium of communicating molecules, namely proteins, lipids and nucleic acids and glycoconjugates. This results in early functional consequences which are independent of lymphocytic infiltration. Concurrently, platelets drive the generation of autoreactive T cells in the peripheral circulation. This presumably occurs by degranulation of platelets immediately upon BBB disturbance, resulting in the release of multiple soluble factors serotonin (5HT), PF4 and PAF, which specifically stimulate differentiation of T cells toward pathogenic Th1, Th17, and IFN-γ/IL-17-producing CD4 T cells (107). However, inflammatory infiltration into the CNS parenchyma occurs subsequently to that of platelets, but for unknown reasons remains less significant in gray relative to white matter. These are significant observations which highlight the multi-modal participation of platelets in the orchestration of neuroinflammation. The question that arises, therefore, is: what are the processes via which platelet responses are linked to the complex and as yet, unelucidated interactions between inflammation and neurodegeneration in MS, which potentially involves multiple immune cell types and their products?

## Potential Pathways for Platelet Involvement in MS

Under inflammatory conditions, platelets bind to other platelets (aggregate) and multiple immune cell types. The interplay between platelets, endothelial cells and leukocytes is the direct cause of BBB damage (108). Platelet activation results within seconds in the expression of surface CD40L and cytokines, notably IL-1ß, leading to endothelial cell expression of adhesion molecules ICAM-1, vascular cell adhesion molecule-1 (VCAM-1), E-selectin and P-selectin (CD62P). IL-1ß release promotes endothelial cell permeability, together with recruitment and attachment, on the endothelium, of several classes of leukocytes, including neutrophils, monocytes, dendritic cells and B and T lymphocytes. Monocytes and neutrophils are the major leukocytes that form complexes with platelets, via P-selectin/PSGL-1 and CD40L/CD40 interactions, leading to monocyte differentiation into dendritic cells and concomitant polymorphonuclear cell activation. Platelet-neutrophil interactions also directly modulate dendritic cell maturation by CD40L/CD40, leading to dendritic cell antigen presentation to T cells. CD40L expression on platelets also enhance CD8^+^ T cell responses, as well as isotype switching in B cells from IgM to IgG. Finally, interactions between platelets and their cellular partners are bi-directional, resulting in amplification and expansion of the inflammatory response (54, 108–112). Thus, platelets have evolved a range of mechanisms resulting in an extensive functional repertoire enabling signaling to multiple immune cell subsets. These mechanisms underlie the evolution of innate to adaptive immunity, thereby placing platelets in a central position for the development of inflammation and autoimmune disease.

Are there any data in the literature that would suggest early platelet involvement in MS? Evidence has been mounting for a vascular component in MS, whereby vascular abnormalities play a crucial role in lesion formation and progression (113). The evidence for this notion comes from reports of enhanced risk of cardiovascular events, such as ischemic stroke, myocardial infarction, and thrombosis in MS patients, directly associated with a dysfunctional coagulation cascade and aberrant platelet function and their increased pro-thrombotic activity (54, 114). Vice versa, platelet defects are known to be a risk factor in MS. For example, comparison of 165 Japanese patients diagnosed with clinically definite MS and 245 healthy controls demonstrated that the frequency of the missense mutation A224D, which impairs PAF-PAF receptor (PAFR) signaling, was significantly higher in MS patients (21.0%) than in healthy controls (13.5%). PAF has been intensely investigated for its proinflammatory response to various stimuli. However not all PAF effects are proinflammatory, since PAF is also involved in immunosuppressive mechanisms and consequently, mutations affecting PAF-PAFR signaling may enhance susceptibility to MS in some patients (115). Additional evidence is provided by the demonstration of an association between MS and mitochondrial mutations (most commonly in Complex 1 components), affecting platelet function. In one case study where a large mitochondrial DNA mutation was identified, a patient diagnosed with myopathy and progressive external ophthalmoplegia (PEO, a condition characterized by eye muscle weakness) also exhibited MS-like features. The mutation caused respiratory chain deficiency in muscle and blood, together with reduced basal platelet mitochondrial membrane potential. However, cerebrospinal fluid analysis and MRI revealed inflammatory CNS demyelination indistinguishable from MS (116) while magnetic resonance spectroscopy showed absence of a significant lactate peak. This was a clear indication that the predominant pathology underlying the MRI data resulted from immune-mediated disease rather than hypoxic/ischemic mechanisms secondary to mitochondrial energy deficits. Thus, the mitochondrial mutation and platelet abnormalities were associated with MS, as well as myopathy and PEO. In this context it is important to remember that mitochondrial mutations and consequential compromised energy supply would also render the high energy-demanding neurons as well as glial cells more susceptible to apoptosis, thereby contributing to progressive disability. The coincidence of an MS-like phenotype with a primary mitochondrial defect (mitochondrial or genomic DNA encoded) suggests that some MS cases may be associated with multisystemic diffuse mitochondrial abnormality (117). Such cases have been reported in Harding's disease, where MS-like disease co-exists with Leber's hereditary optic neuropathy (LHON), and others where the primary mitochondrial disorder was identified as a mutation in the optic atrophy 1 (*OPA1*) gene, or the mitochondrial DNA polymerase gamma (*POLG1*) gene.

An alternative mechanism is suggested by recent findings on the role of the commensal microbiome in autoimmune disease and the gut-brain axis. There is now increasing evidence that dysbiosis (or reduction or loss of normally-residing gut microbiota) constitute an environmental factor, which can modulate immune processes relevant to MS by boosting the polarization of proinflammatory cells (118, 119). Reduced diversity of microbiota has been documented in MS patients with active disease. For example, *Parabactoroides distasonis*, which is frequently found to be decreased in MS patients enhances the differentiation of IL-10^+^ Tregs. On the other hand, abundance of *Akkermensia municiphilia* boosts Th1 and Th17 differentiation, while suppressing that of Treg. Gut T cells traffic to lymphoid tissues and the CNS where they can have direct effects on inflammation. Studies by Linden et al. and Rumah et al. (120, 121) showed that toxic products from gut bacteria may initiate new lesion formation. They demonstrated that the gut bacterium *Clostridium perfringes* releases the epsilon protein which may play a pivotal role in triggering new lesions due to its tropism for myelin and the BBB. Epsilon toxin is a 33 KD precursor cleaved in the gut to a 28.6 KD product. In mammalian brain slices, cleaved epsilon toxin has been shown to bind myelin. It also has the capacity to enter the blood stream and bind to CNS microvessel endothelial cells forming a pore on the endothelial plasma membrane, thereby compromising BBB integrity. Such disruption of the BBB would have an immediate effect on platelet homeostasis, resulting in platelet activation, platelet adhesion/aggregation on the vascular surface and platelet interactions with multiple immune subsets, as described above, resulting in an inflammatory response and lesion development. A relationship has recently been demonstrated between platelet counts and alterations in the gut microbiome, whereby the reduction in “good” microorganisms is inversely related to platelet numbers (122). Overall, gut microbiome-directed therapeutic strategies based on microbiome profiling in patients with MS and in the EAE mouse model have attracted major interest, however, systematic clinical trials are yet outstanding (123).

It has also been documented that oligodendrocyte death appears to be an initial event in lesion development. Thus in the study of Lucchinetti et al. (25) already described, Type III lesions were characterized by oligodendrocyte loss ahead of demyelination, whereas T cell infiltration was mild until large scale demyelination became evident. Similarly, in a study of new symptomatic lesions in patients who died shortly after a relapse, Barnett and Prineas (124) identified severe oligodendrocyte apoptosis, associated with reactive microglia, but few myelin-laden macrophages and essentially intact myelin. Strikingly, this occurred in the absence or minimal evidence of lymphocytic infiltration. Following oligodendrocyte, death large amounts of membrane become metabolically unsupported, with ensuing accumulation of myelin fragments and vesicles at the site of the early lesion. Therefore it can be envisaged that these events would impact the BBB, if the capacity of macrophages and microglia to remove myelin debris became exceeded (125).

Any of the above proposed scenarios, singly or in combination, would represent a candidate mechanism potentially triggering platelet involvement in disease development. For example:

(a) Steady release of toxic products from gut bacteria into the circulation would lead to failure to maintain vascular integrity and to compromised BBB function. This would be met with immediate platelet activation, platelet association with the endothelial layer and platelet inflammatory cascades.
(b) Vascular damage and extravascular accumulation of blood components, especially fibrin/fibrinogen is believed to drive local inflammation resulting in microglial activity, release of pro-inflammatory chemokines across a leaky BBB and recruitment of peripheral macrophages, again resulting in platelet activation.
(c) Myelin debris, for example by oligodendrocyte death or local inflammation resulting from fibrinogen accumulation first collect in the interstitial fluid. This is followed by migration of antigens into perivascular channels, eventually accumulating in lymph nodes via soluble and cellular routes. Alternatively, antigens are collected by dendritic cells within the CNS parenchyma and travel to lymph nodes. Antigens are then presented to CD4^+^ and CD8^+^ cells which enter the CNS, infiltrate the cerebrospinal fluid (CSF) entering the circulation via a damaged BBB, where they encounter platelets (125).

This diversity of mechanisms and myelin products which can be recognized as antigens are also in keeping with the heterogeneity of the disease.

## The Potential of Platelets as Biomarkers and Therapeutic Targets in MS

MRI remains the gold standard for the diagnosis of MS and for patient monitoring. Nonetheless, MRI has a number of major limitations in the context of MS, such as discordance between lesion location and clinical presentation, as well as low sensitivity of conventional approaches to cortical lesions and diffuse white matter damage (126). Novel and often very advanced imaging approaches are continuously being explored to improve early diagnosis and better identification of MS phenotypes. This includes the combination of MRI with nuclear medicine imaging modalities, such as positron emission tomography (PET) and single photon emission computed tomography (SPECT), whereby MRI provides anatomical information about lesion topography and lesion load, while nuclear imaging can identify physiological changes at the single cell or single molecule level. For example, nuclear imaging represents an attractive candidate modality for improved detection and further characterization of cortical lesions. Accumulation of platelets at the lesion site is a very plausible hypothesis based on several potential mechanisms. (1) Meningeal accumulation of B and T cells via leakage of the BBB. It is known that platelets form aggregates with various immune cells and thus are transported via piggybacking (48, 50, 54). (2) Platelets have been shown to migrate and thus may associate with neurons (84, 100, 101). In the following paragraphs we will discuss improvements in nuclear medicine imaging and the potential for identification of adjunct biomarkers and molecular targets in MS.

Early platelet involvement in MS can potentially provide a unique opportunity to use platelets as biomarkers for diagnostic, therapeutic and combined theranostic approaches. Direct targeting of specific biomarkers for the diagnosis of MS has been demonstrated using the peripheral benzodiazepine receptor, also known as TSPO, a protein that is only minimally expressed in healthy brain (127). Currently more than 80 TSPO radiotracers are under development for molecular positron emission tomography imaging, with the aim to improve signal to background ratio, as well as to overcome low binding status in over 30% of the population with a single nucleotide polymorphism (127). Other molecular imaging approaches for MS include the targeting of adenosine receptors, which are key element in inflammation and thrombosis (128). These promising approaches are demonstrating that molecular imaging targeting specific markers has the potential to provide specific and early imaging of MS. We propose that molecular markers specific for activated platelets will be worth to be tested as novel diagnostic approach for the imaging and diagnosis of MS.

Amongst the various receptors expressed on platelets, P-selectin and GPIIb/IIIa receptors are the most often used biomarkers because they enable the differentiation between resting and activated platelets. Upon platelet activation, P-selectin expression is upregulated by translocation from the intracellular granules to the external membrane. P-selectin is, however, not platelet-specific because of its expression on other cells, including endothelial cells. The GPIIb/IIIa receptor, on the other hand, is only found on platelets and undergoes a conformational change during platelet activation (129). This exposes activation-specific epitopes, such as ligand-induced binding sites and the ligand (fibrinogen) binding pocket. In addition to this activation and platelet specificity, GPIIb/IIIa is highly abundant with about 60,000 receptor molecules/platelet. As such small numbers of platelets expose abundant molecular target epitopes, which is an important advantage toward a superior sensitivity of the respective molecular imaging. These features make GPllb/IIIa an ideal epitope for molecular targeting.

Across the cardiovascular and cancer fields, technologies used for the molecular imaging of platelets include ultrasound (130–134), positron emission tomography [PET (134, 135)], single photon emission computed tomography [SPECT (136, 137)] and optical imaging (136, 137). Each of these modalities have their strengths and weaknesses (138–142), which have to be assessed to determine their suitability for diagnosing MS. In particular, MRI has been commonly used for the detection of MS lesions (143), therefore the addition of molecular imaging to detect activated platelets may enable earlier and more sensitive diagnosis. Since the current imaging methods provide a readout only after anatomical/functional changes have occurred, molecular imaging of activated platelets might allow us to improve the specificity of MS diagnosis, even in areas of small lesions. Furthermore, activated platelet imaging might represent an approach for longitudinal monitoring of MS progression and provide the ability for the monitoring of treatment success or failure.

A wide selection of platelet targeting ligands have been conjugated onto contrast agents for the respective imaging technologies, and have provided direct *in vivo* visualization of platelets as major components of thrombi (128). These studies are helpful in directing us to the platelet contrast agent best suitable for the diagnosis of MS. Using fucodian (a sialyl Lewis X mimetic with a strong affinity to P-selectin), Letourneur et al. successfully imaged platelets *in vivo* in arterial thrombi via SPECT (144) and via MRI (138). More recently, Jing et al. conjugated microbubbles with commercially available antibodies that target P-selectin for *in vivo* molecular ultrasound imaging of platelets in left atrial thrombi in rats (138, 145). Arginine-glycine-aspartic acid (RGD) analogs have been used for visualization of platelets in vascular thrombosis via a range of ultrasound, MRI and nuclear imaging (129, 139, 146). However, RGD is not specific for the GPIIb/IIIa integrin and indeed has been used for the targeting of other integrins, such as the vitronectin receptor αvβ3 in cancer imaging (128). Furthermore, RGD analogs are not specific for activated platelets.

More interestingly, GPIIb/IIIa antagonists, such as elarofiban and abxicimab (147–149) have been employed as ligands for imaging. However, these antagonists similar to RGD analogs bind to all circulating platelets, thereby specificity of their diagnosis has been questioned. Peter and colleagues have generated single-chain antibodies (scFv) with specific targeting toward the activated GPIIb/IIIa integrin, and no binding to resting platelets in circulation (150). This scFv have been conjugated to microbubbles for ultrasound (151), iron oxides particles or perfluorocarbon nanoemulsions for MRI (131), radiotracers for PET (137) and near-infrared dyes for optical imaging (137) of arteria and venous thrombi. Using this scFv, Yap et al. successfully visualized a board range of tumors via ultrasound, PET and optical imaging (133). Additionally, Yap et al. conjugated this scFv with a potent chemo-therapeutic microtubule inhibitor and demonstrated that these antibody-drug conjugates could prevent tumor growth and metastases (133). This concept of activated platelet targeting may be transferable and seems highly attractive as a diagnostic tool as well as targeted, site-specific therapy for MS. The latter promises to achieve high lesion-localized drug accumulation with low system drug concentrations.

As mentioned in the previous section, the depletion of platelets resulted in the elimination of EAE progression. Clinically, anti-platelet therapies including the cyclooxygenase 1 inhibitors aspirin, P_2_Y_12_ receptor inhibitors, PAR1 antagonists, and GPIIb/IIIa inhibitors, are used for the prevention of platelet aggregation. As such, multiple anti-platelet therapies would be available for the potential treatment of MS patients. The activated-platelet targeting approach of CD39 has been successfully tested in cardiac ischemia/reperfusion injury in mice. In this setting the anti-inflammatory effect of the platelet-targeted construct contributed directly to the prevention of ischemia/reperfusion injury. Similarly, this construct would use platelets as targets to direct and accumulate the anti-inflammatory effect to lesion sites.

Preclinical research into targeted drug delivery systems and nano-/micro-carriers have been extensively conducted to overcome bleeding complications, and the employment of these side-effect free drugs may help to successfully treat MS. With genetic engineering of recombinant antibody-fusion drugs, scFvs targeting activated GPIIb/IIIa have been paired with/conjugated to several drugs, including urokinase-type plasminogen activator (134), tick anticoagulation peptide (152, 153), and CD39 (ectonucleoside triphosphate diphosphohydrolase-1) (154). These antibody-fusion drugs have been used successfully for side-directed low systemic dose treatment and prevention of thrombosis, myocardial ischemic/reperfusion injury and sepsis. Of the three different drugs, the scFv-CD39 seems to be the most ideal drug candidate to stop MS progression, because CD39 is both anti-thrombotic and anti-inflammatory (155, 156). It is known that inflammation plays an important role in MS and adenosine receptors have been proven to be upregulated in the area of MS (128–130). Therefore, the dual action of anti-platelet targeting of the scFv, together with the anti-inflammatory NTPase breakdown of adenosine by the CD39 component, will likely play a beneficial role in the treatment of MS. Respective preclinical studies are currently ongoing.

## Conclusions

Historic and more recently emerging evidence indicate an early and important role of platelets in MS. This includes the consistent demonstration of platelet abnormalities and release of platelet inflammatory products in MS patients and of presence of platelet products in MS post-mortem lesions. Some of these biochemical and ultrastructural defects were identified from the CIS stage. Preclinical data generated proof of concept that platelet depletion prevents the development of EAE in mice and that early platelet infiltration into the CNS parenchyma is neurodegenerative. However, the direct translation of these findings into the use of anti-platelet drugs in patients with MS is clearly impeded by an increased bleeding risk, generally associated with the use of currently available anti-platelet drugs. On the other hand, activated platelets may provide a unique molecular epitope for early diagnosis of MS and for therapeutic drug-targeting, the latter potentially avoiding bleeding complications. As such we hypothesize that platelet targeting for diagnosis and therapy holds great promise and warrants further investigations.

## Dedication

This work is dedicated to the memory of Christine Heim.

## Author Contributions

CD'S, PK, MH, and JO generated the data based on the EAE model discussed in section An Association Between Platelets and MS/EAE. XW contributed to the imaging data discussed in section The Potential of Platelets as Biomarkers and Therapeutic Targets in MS. GK provided the advice on the translational aspects of the research. JO wrote the sections Introduction to The Potential of Platelets as Biomarkers and Therapeutic Targets in MS. XW and KP wrote the section The Potential of Platelets as Biomarkers and Therapeutic Targets in MS. KP wrote the section Conclusions of the manuscript. JO and KP reviewed the manuscript. All authors contributed to the article and approved the submitted version.

## Conflict of Interest

JO, XW, and KP are inventors on a patent application on diagnosis and therapy of MS. The remaining authors declare that the research was conducted in the absence of any commercial or financial relationships that could be construed as a potential conflict of interest.
